# Mitochondrial Quality Control Governed by Ubiquitin

**DOI:** 10.3389/fcell.2020.00270

**Published:** 2020-04-24

**Authors:** Sonia Ravanelli, Fabian den Brave, Thorsten Hoppe

**Affiliations:** ^1^Institute for Genetics and Cologne Excellence Cluster on Cellular Stress Responses in Aging-Associated Diseases, University of Cologne, Cologne, Germany; ^2^Department of Molecular Cell Biology, Max Planck Institute of Biochemistry, Martinsried, Germany; ^3^Center for Molecular Medicine Cologne, University of Cologne, Cologne, Germany

**Keywords:** *C. elegans*, mitochondria, proteostasis, mitochondria-associated degradation (MAD), ubiquitin, Cdc48, p97, Msp1

## Abstract

Mitochondria are essential organelles important for energy production, proliferation, and cell death. Biogenesis, homeostasis, and degradation of this organelle are tightly controlled to match cellular needs and counteract chronic stress conditions. Despite providing their own DNA, the vast majority of mitochondrial proteins are encoded in the nucleus, synthesized by cytosolic ribosomes, and subsequently imported into different mitochondrial compartments. The integrity of the mitochondrial proteome is permanently challenged by defects in folding, transport, and turnover of mitochondrial proteins. Therefore, damaged proteins are constantly sequestered from the outer mitochondrial membrane and targeted for proteasomal degradation in the cytosol via mitochondrial-associated degradation (MAD). Recent studies identified specialized quality control mechanisms important to decrease mislocalized proteins, which affect the mitochondrial import machinery. Interestingly, central factors of these ubiquitin-dependent pathways are shared with the ER-associated degradation (ERAD) machinery, indicating close collaboration between both tubular organelles. Here, we summarize recently described cellular stress response mechanisms, which are triggered by defects in mitochondrial protein import and quality control. Moreover, we discuss how ubiquitin-dependent degradation is integrated with cytosolic stress responses, particularly focused on the crosstalk between MAD and ERAD.

## Introduction

Mitochondrial integrity relies on a sophisticated network of quality control machineries, which have been evolved to counteract challenges associated with the endosymbiotic integration of this organelle into eukaryotic cells (Youle, 2019). Along with a precise coordination between nuclear and mitochondrial gene expression (Couvillion et al., 2016), transcriptional stress response programs emerged as central mitochondrial surveillance mechanisms (Andréasson et al., 2019). Mitochondrial functionality is further supported by ubiquitin-dependent degradation of proteins accumulating under stress conditions. The outer and inner mitochondrial membrane (OMM and IMM) separate the lumen into the intermembrane space (IMS) and the matrix. Mitochondria are equipped with an elaborate set of proteases acting on the different sub-compartments, to maintain the mitochondrial proteome from the inside (Koppen and Langer, 2007; Quirós et al., 2015; Glynn, 2017). Otherwise, the integrity of the mitochondrial proteome is largely supported by the UPS localized in the cytosol (Franz et al., 2015; Bragoszewski et al., 2017; Braun and Westermann, 2017; D’Amico et al., 2017; Escobar-Henriques et al., 2020).

Besides the important role in ATP production and synthesis of amino acids, nucleotides, and iron-sulfur clusters (Lill and Mühlenhoff, 2008), mitochondria are also required for calcium buffering and apoptosis regulation (Wang and Youle, 2009; Contreras et al., 2010). Notably, mitochondria are tightly interconnected with other cellular organelles, especially the endoplasmic reticulum (ER) (Helle et al., 2013; van Vliet et al., 2014; Phillips and Voeltz, 2016). The contact between ER and mitochondria has been associated with aging and age-related diseases (Moltedo et al., 2019). In fact, the physical interaction between these tubular organelles supports the transfer of lipids, calcium ions and other metabolites, localizes to DNA nucleoids and regulates mitochondrial dynamics (Helle et al., 2013; Raturi and Simmen, 2013; van Vliet et al., 2014; Phillips and Voeltz, 2016; Moltedo et al., 2019). Recent studies suggest an intricate cooperation between ER and mitochondria in proteostasis (Dederer et al., 2019; Matsumoto et al., 2019), which constitute a newly developing research field promising for the development of therapeutic interventions as proposed for cardiac pathologies (Arrieta et al., 2020).

In this review we focus on quality control pathways that maintain mitochondrial functionality, involving cross-communication with the ER and other cellular compartments. We discuss recent discoveries on the role of the ubiquitin/proteasome-system (UPS) in mitochondrial quality control, including pathways that are constitutively active or triggered by metabolic stress to ensure mitochondrial integrity.

## Protein Degradation Mechanisms

Protein quality control is required at all steps originating from protein synthesis and involves a series of mechanisms dedicated to the surveillance of protein translation, transport, and turnover (Kaushik and Cuervo, 2015). Central players of the proteostasis network are molecular chaperones which mediate folding, targeting, and degradation of proteins (Klaips et al., 2018). The two major degradation pathways for proteins in the cytosol are the UPS and the autophagy-lysosomal pathway (Pohl and Dikic, 2019). In both proteolytic systems post-translational attachment of the small polypeptide ubiquitin serves as a targeting signal for protein turnover. The modification with ubiquitin (ubiquitylation) is mediated by a three-step enzymatic cascade (Kerscher et al., 2006). First, the ubiquitin-activating enzyme (E1) forms a high-energy thioester bond between its catalytic cysteine and the C-terminal glycine residue of ubiquitin, which is then transferred to a cysteine of an ubiquitin-conjugating enzyme (E2). The E2 cooperates with specific ubiquitin ligases (E3) to mediate the covalent attachment of ubiquitin mainly to a lysine residue in the selected substrate. Repeated cycles of this reaction either results in multiple mono-ubiquitylation of different lysine residues of a given substrate or formation of ubiquitin chains by targeting one of the seven lysines of ubiquitin (K6, K11, K27, K29, K33, K48, or K63) (Haakonsen and Rape, 2019). These different ubiquitin-dependent modifications termed “the ubiquitin code” serve as signals for different downstream events mediated by specialized binding proteins. Moreover, ubiquitylation can be reversed by different deubiquitylating enzymes (DUBs), which completely remove ubiquitin from substrates or trim ubiquitin chains to alter their composition (Clague et al., 2019). A prominent role of ubiquitylation is to target proteins for lysosomal or proteasomal turnover, which is often mediated by attachment of K48-linked ubiquitin chains (Dikic, 2017).

The turnover of soluble ubiquitylated proteins is mainly conducted by the 26S proteasome (Bard et al., 2018). It constitutes a multicatalytic complex of a barrel-shaped core subunit known as 20S proteasome and the 19S regulatory particle, attached to one or both ends of the 20S core. The regulatory particle is composed of a hexameric complex of AAA-ATPases that coordinates unfolding and translocation of the substrate into the core subunit. The 19S subunit also contains scaffold proteins involved in substrate recognition and deubiquitylation as well as gate opening and binding with other external factors. The core subunit is composed of four stacked heptameric rings and contains the proteolytic activity required for the cleavage of unfolded polypeptides.

In contrast to soluble substrates, proteins organized in multimeric complexes or membrane bound, require an additional extraction step prior to proteasomal degradation. This function is mainly executed by Cdc48 (p97 or VCP in vertebrates), which belongs to the family of AAA-ATPases associated with diverse cellular activities (AAA+), commonly using ATP to perform mechanochemical reactions (Sauer and Baker, 2011; Glynn, 2017). In collaboration with substrate-specific cofactors, Cdc48 binds ubiquitylated proteins and targets them to the 26S proteasome (Franz et al., 2014; Barthelme and Sauer, 2016).

Larger structures such as aggregated proteins or organelles are targeted for degradation inside lysosomes (vacuole in yeast and plants), which contain promiscuous proteolytic enzymes for degradation of engulfed cargoes. In this process termed macroautophagy (hereafter autophagy), substrates are recognized by autophagy receptors bound to autophagosomal membranes, which triggers substrate engulfment by the autophagosomal membrane and subsequent lysosomal fusion (Khaminets et al., 2016). Similar to soluble misfolded proteins, protein aggregates are targeted for autophagic degradation by ubiquitylation, which is a common feature of the two proteolytic pathways (Lu et al., 2017). Moreover, a specialized form of autophagy, called mitophagy, allows the selective turnover of entire mitochondria. This process can either be mediated by autophagy receptors residing in the OMM or by ubiquitylation of OMM proteins (Palikaras et al., 2018; Pickles et al., 2018).

## Ubiquitin-Dependent Surveillance of the Mitochondrial Proteome

The degradation of ER-resident or mitochondrial proteins is regulated by two mechanistically similar ubiquitin-dependent pathways termed ER-associated degradation (ERAD) and mitochondria-associated degradation (MAD) (Heo and Rutter, 2011; Guerriero and Brodsky, 2012; Ruggiano et al., 2014; Braun and Westermann, 2017; Mehrtash and Hochstrasser, 2018). For both tubular organelles, proteins residing in the outer membrane are directly accessible for ubiquitylation. By contrast, proteins localized inside these organelles have to be transported across the membrane to be exposed to the ubiquitin-conjugation machinery. Cdc48 triggers ERAD by retro-translocation of substrate proteins out of the ER lumen and proteasomal degradation at the cytosolic side of the ER membrane. Therefore, substrates proteins are ubiquitylated by membrane-bound ubiquitin ligase complexes. These ERAD ligases interact with accessory factors such as the UBX-domain protein Ubx2 to recruit Cdc48 for coordinating substrate extraction and turnover (Neuber et al., 2005; Schuberth and Buchberger, 2005). Increasing evidence suggests that a comparable system employs the UPS in controlling quality control of mitochondrial proteins.

### UPS-Dependent Turnover of Mitochondrial Proteins

**FIGURE 1:**
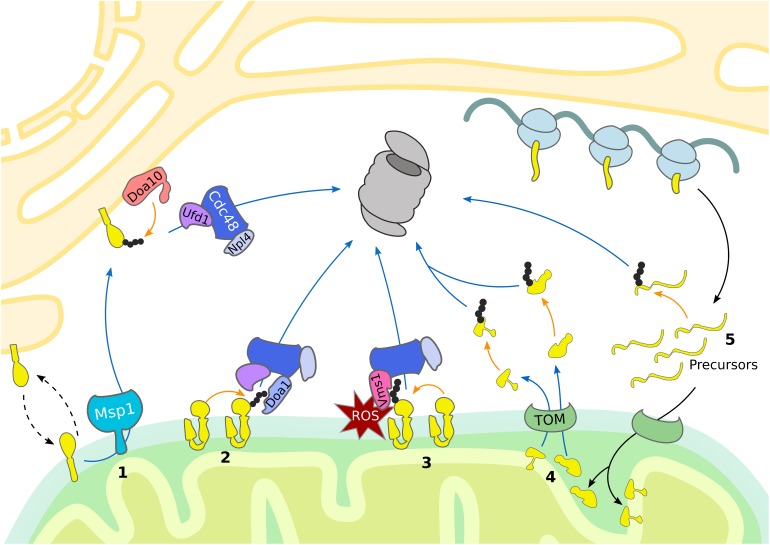
UPS-dependent turnover of mitochondrial proteins. The cytosolic UPS mediates mitochondrial protein turnover by ubiquitylation (orange arrows) and targeting (blue arrows) of substrates for degradation by the 26S proteasome. (1) Mislocalized tail-anchored proteins are extracted from the OMM by Msp1, ubiquitylated by the ER-associated E3 ubiquitin ligase Doa10 and then translocated to the proteasome by the Cdc48^Ufd1/Npl4^ complex. (2, 3) Degradation of OMM proteins occurs via Cdc48-dependent translocation to the 26S proteasome. (2) Upon oxidative stress, Vms1 translocates to the OMM where it recruits Cdc48 and its co-factor Npl4. (3) Under normal conditions OMM proteins are ubiquitylated and translocated to the 26S proteasome by Cdc48 together with the co-factors Ufd1, Npl4, and Doa1/Ufd3. (4) Proteins residing in the IMS and IMM are retro-translocated via the TOM complex into the cytosol for ubiquitin-dependent proteasomal degradation. (5) Prior import, mistargeted or damaged mitochondrial precursor proteins are degraded by the UPS.

The mitochondrial mass is efficiently regulated by the balanced coordination between biogenesis and degradation. In addition to the degradation of damaged mitochondrial proteins the UPS also contributes to remodeling of the mitochondrial proteome in response to metabolic changes (Figure 1) (Bragoszewski et al., 2017). Mitochondria form a highly dynamic network shaped by continuous fusion and fission events with adaptive morphology according to cellular needs. These fission and fusion events are mediated by large dynamin-like GTPases that provide the mechanical force to either fuse or separate membranes. In mammals, fission is induced by DRP1 (Dnm1 in yeast), whereas fusion is driven by mitofusins (Fzo1 in yeast) in the outer membrane and by OPA1 (Mgm1 in yeast) in the inner membrane (Escobar-Henriques and Anton, 2013). Fusion is known to enhance the exchange of important molecules and can temporarily compensate for defects in mitochondrial sub-populations. On the other hand, fission promotes mitochondrial motility and allows separation of damaged organelles for mitophagy (Lackner, 2014; Roy et al., 2015; Wai and Langer, 2016). Residing in the OMM, these GTPases present cytosolic domains which are targeted for ubiquitin-dependent degradation. Thus, the UPS provides a crucial role in the regulation of mitochondrial morphology and function (Bragoszewski et al., 2017).

Notably, the first identified MAD substrate was in fact the yeast mitofusin Fzo1 (Neutzner and Youle, 2005), which is embedded into the OMM by two transmembrane domains, exposing most of its amino acid residues to the cytosol. Ubiquitylation of Fzo1 mainly depends on the F-box protein Mdm30, which is part of the multi-subunit SCF (Skp, Cullin, Fbox) ubiquitin ligase complex (Fritz et al., 2003; Escobar-Henriques et al., 2006; Cohen et al., 2008) (Table 1). The ubiquitin-modification of Fzo1 provides a regulatory role in the fusion of mitochondrial outer membranes, which is not necessarily linked to degradation (Anton et al., 2011; Cohen et al., 2011). In fact, proteasome-dependent turnover of Fzo1 largely depends on the nature of ubiquitylation, since the DUB Ubp2 is able to remove or trim down ubiquitin chains that are attached to Fzo1, which regulates its degradation (Anton et al., 2013) (Table 1).

**TABLE 1:** Regulators of ubiquitin-dependent mitochondrial quality control.

**Function**	**Yeast**	**Mammals Other species**	**Function**	**Pathways**	**Reference**
Translocation	Cdc48	VCP/p97 *cdc-48* (*C. elegans*) TERT94/VCP (*D. melanogaster*)	AAA-ATPase	ERAD, MAD, RQC, mitoTAD	Heo et al., 2010; Tanaka et al., 2010; Xu et al., 2011; Brandman et al., 2012; Defenouillère et al., 2013; Kim et al., 2013; Wang et al., 2016; Wu et al., 2016; Izawa et al., 2017; Rendón et al., 2018; Verma et al., 2018; Mårtensson et al., 2019; Matsumoto et al., 2019
	Vms1	VMS1/ANKZF1 *vms-1* (*C. elegans*)	Cdc48 recruitment	MAD, RQC	Heo et al., 2010; Izawa et al., 2017; Rendón et al., 2018; Verma et al., 2018
	Ubx2		Cdc48 recruitment	ERAD, mitoTAD	Mårtensson et al., 2019; Matsumoto et al., 2019
	Doa1/Ufd3		Cdc48 co-factor	MAD	Wu et al., 2016
	Ufd1	UFD1L	Cdc48 co-factor	MAD, ERAD, mitoTAD, RQC	Brandman et al., 2012; Wu et al., 2016; Izawa et al., 2017; Verma et al., 2018; Mårtensson et al., 2019; Matsumoto et al., 2019
	Npl4	NPL4	Cdc48 co-factor	MAD, ERAD, mitoTAD, RQC	Heo et al., 2010; Brandman et al., 2012; Wu et al., 2016; Izawa et al., 2017; Verma et al., 2018; Mårtensson et al., 2019; Matsumoto et al., 2019
	Msp1	ATAD1	AAA-ATPase	MAD, mitoCPR	Chen et al., 2014; Okreglak and Walter, 2014; Weidberg and Amon, 2018; Dederer et al., 2019; Matsumoto et al., 2019
	Cis1		Msp1 recruitment	mitoCPR	Weidberg and Amon, 2018
		Ubiquilins			Itakura et al., 2016; Whiteley et al., 2017
Ubiquitylation	Mdm30		E3 ligase	MAD	Fritz et al., 2003; Escobar-Henriques et al., 2006; Cohen et al., 2008
	Rsp5		E3 ligase	MAD	Wu et al., 2016
		Parkin	E3 ligase	MAD	Tanaka et al., 2010; Chan et al., 2011; Kim et al., 2013; Wang et al., 2016
	Ltn1	Listerin	E3 ligase	RQC	Brandman et al., 2012; Defenouillère et al., 2013; Shao et al., 2013
		MARCH5/MITOL	E3 ligase	MAD	Yonashiro et al., 2006; Karbowski et al., 2007; Park et al., 2010
	Doa10		E3 ligase	ERAD	Dederer et al., 2019; Matsumoto et al., 2019
	Cue1		Doa10 co-factor	ERAD	Dederer et al., 2019; Matsumoto et al., 2019
	Ubc6		E2	ERAD	Dederer et al., 2019; Matsumoto et al., 2019
	Ubc7		E2	ERAD	Dederer et al., 2019; Matsumoto et al., 2019
De-ubiquitylation	Ubp2		DUB	MAD	Anton et al., 2013
Transcriptional regulation	Pdr3		Transcription factor	mitoCPR	Weidberg and Amon, 2018
	Rpn4		Transcription factor	Response to clogging	Boos et al., 2019
		ATF5 *atfs-1 (C. elegans)*	Transcription factor	UPR^*mt*^	Nargund et al., 2012; Fiorese et al., 2016
		ERα	Transcription factor	UPR^*mt*^	Papa and Germain, 2011
	Msn2/4		Transcription factor	IPTP	Suhm et al., 2018
					

Depending on the physiological conditions, Cdc48 also appears to be required for Fzo1 regulation (Heo et al., 2010; Esaki and Ogura, 2012). Under oxidative stress conditions, Cdc48 together with its co-factors Vms1 and Npl4 facilitates Fzo1 degradation (Heo et al., 2010). Under non-stressed conditions Fzo1 and additional other OMM proteins are targeted by another Cdc48 complex involving Npl4, Ufd1, and Doa1, also called Ufd3 (Wu et al., 2016) (Figure 1 and Table 1). This might however reflect protein quality control triggered by experimental tagging of the membrane-bound proteins, since non-tagged endogenous Fzo1 is rather stabilized by Cdc48, which exerts a regulatory role during OMM fusion (Simões et al., 2018; Anton et al., 2019). Turnover of mitofusins by MAD is widely conserved among species including Marf in flies (Ziviani et al., 2010; Wang et al., 2016) and Mfn1/2 in mammals (Tanaka et al., 2010; Chan et al., 2011; Xu et al., 2011). A common role of MAD in this case is the inhibition of mitochondrial fusion by mitofusin degradation, resulting in mitochondrial fragmentation. In mammals, the E3 ligases Parkin and MARCH5 mediate p97 dependent extraction and proteasomal turnover of mitofusins, inducing mitochondrial fission and mitophagy (Karbowski et al., 2007; Ziviani et al., 2010; Chan et al., 2011; Xu et al., 2011; Wang et al., 2016). Moreover, MAD was observed to play a role in regulation of apoptosis, since it targets the anti-apoptotic BCL2 protein MCL1 for degradation in mammals (Inuzuka et al., 2011; Xu et al., 2011).

Besides Cdc48, Msp1 (ATAD1 in humans) supports extraction and degradation of mitochondrial proteins (Figure 1 and Table 1). Despite being an AAA-ATPase similar to Cdc48, Msp1 contains a membrane spanning domain at the N-terminus and is mainly localized at the OMM, but is also attached to peroxisomes (Nakai et al., 1993; Chen et al., 2014; Okreglak and Walter, 2014). Msp1/ATAD1 plays a key role in mitochondrial proteostasis since it mediates the degradation of mislocalized tail-anchored proteins (TA-proteins) from the OMM. A baseline degradation of TA-proteins by the UPS occurs to maintain a dynamic stationary level and ensure insertion into the outer membrane of the correct organelle (Chen et al., 2014; Okreglak and Walter, 2014). Moreover, TA-proteins destined to the ER can mislocalize to the OMM, when their import system, termed the GET pathway in yeast and TRC in mammals, is impaired. These mislocalized TA-proteins are recognized by Msp1 at the OMM and targeted for proteasomal degradation. For instance, the TA-protein Pex15 is partly inserted in the OMM, even in the presence of the fully functional GET pathway, suggesting that TA-proteins are constitutively inserted into the OMM, where they have to be removed by Msp1 (Chen et al., 2014; Okreglak and Walter, 2014). Recently, a similar role of Msp1 was described at peroxisomes (Weir et al., 2017). Of note, the proposed ATP-dependent activity of Msp1 in the extraction of TA-proteins from mitochondrial membranes was confirmed in an *in vitro* system of reconstituted proteoliposomes (Wohlever et al., 2017).

Surprisingly, mistargeted TA-proteins extracted by Msp1 are ubiquitylated by Doa10, an E3 ligase residing in the ER membrane and degraded in a Cdc48-dependent manner (Figure 1 and Table 1). This initial observation suggests a role of ERAD in the turnover of OMM proteins (Dederer et al., 2019; Matsumoto et al., 2019). Indeed, Msp1 co-localizes with its substrates at ER-mitochondria contact sites, which however, seem to be dispensable for Doa10/Cdc48 dependent turnover of Msp1 substrates (Matsumoto et al., 2019). Therefore, it was proposed that Msp1 extracted TA-proteins are inserted into the ER membrane for Doa10-mediated ubiquitylation. Subsequently, Cdc48 is recruited together with the cofactors Ufd1 and Npl4 to translocate mistargeted TA-proteins to the 26S proteasome (Matsumoto et al., 2019). Intriguingly, Msp1 appears to extract only monomeric proteins and not multi-complexes, suggesting that the recognition of mistargeted TA-protein is based on the weak interaction with the membrane of a single transmembrane domain (Dederer et al., 2019). Interestingly, Msp1 emerged as an MAD substrate itself, whose degradation depends on the Doa1–Cdc48^–Ufd1–Npl4^ complex (Wu et al., 2016). This might indicate a role of the UPS in the regulation of mitochondrial protein half-life by controlling Msp1 level and extraction of TA-proteins.

Intriguingly, besides membrane bound proteins also inner mitochondrial proteins have been reported to be ubiquitylated. In analogy to ERAD, retro-translocation of mitochondrial proteins across IMM and/or OMM has been suggested to enable ubiquitylation at the cytosolic surface of the organelle (Figure 1). Indeed, such activity has been demonstrated for IMS proteins, which upon unfolding, translocate into the cytosol (Bragoszewski et al., 2015). The export of these proteins seems to depend on the translocase of the outer membrane (TOM) complex also required for import, however, further studies are required to investigate the exact mechanism of mitochondrial retro-translocation. Notably, this degradation of IMS proteins appears to be utilized to rewire the mitochondrial proteome to changing metabolic conditions upon shift from respiration to fermentation (Bragoszewski et al., 2015). A major challenge in exploring this novel mechanism is to carefully distinguish ubiquitin-dependent degradation of retro-translocated mitochondrial protein versus cytosolic precursor proteins that accumulate when mitochondrial import is impaired (Figure 1).

### Mitochondrial Import Control

**FIGURE 2:**
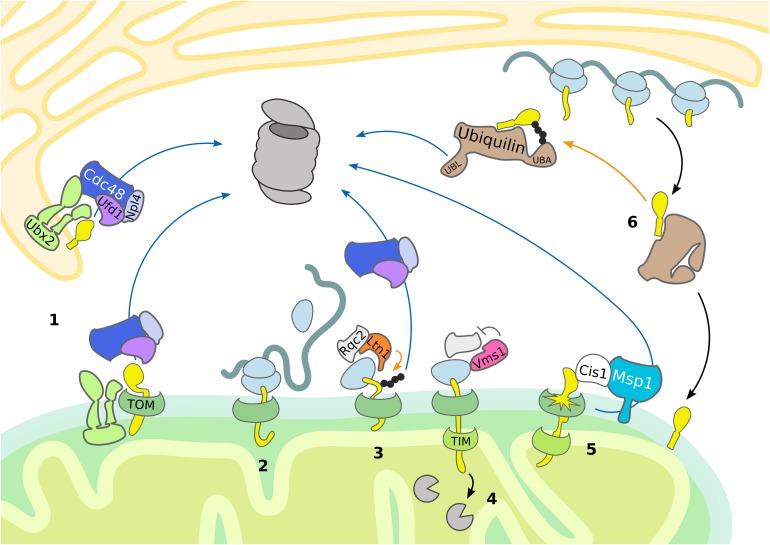
Mitochondrial import control. The cytosolic UPS supports quality control and mitochondrial import by removing damaged proteins. Substrate ubiquitylation (orange arrows) is followed by translocation (blue arrows) and proteasomal degradation. (1) Ubx2 localizes both in the ER membrane and in proximity of the TOM complex at the OMM, where it recruits Cdc48 and its cofactors Ufd1 and Npl4 to degrade ER and mitochondrial proteins, respectively. (2, 3, 4) In case of co-translational import of mitochondrial proteins, induction of RQC is central to the handling of stalled ribosomes. (3) Ltn1-dependent ubiquitylation of the nascent polypeptide chain recruits Cdc48 with its cofactors Ufd1 and Npl4 for proteasomal targeting. (4) Vms1 counteracts Rqc2-dependent CAT-tail formation and safeguards tRNA release of the nascent polypetide, which is subsequently degraded in the matrix by mitochondrial proteases. (5) Upon mitochondrial import defects, Cis1-recruited Msp1 moderates the release of mitochondrial proteins stalled in the TOM complex. (6) In mammals, ubiquilins bind transmembrane domains of mitochondrial proteins and either support mitochondrial translocation or proteasomal targeting.

Nuclear-encoded mitochondrial proteins are synthetized as precursors in the cytosol and subsequently imported and sorted to different mitochondrial compartments. After passing through the TOM complex, precursor proteins are redistributed to their final intra-mitochondrial destination. At least five distinct but interconnected import pathways have been identified, each one directed by a specific targeting signal (Neupert and Herrmann, 2007; Chacinska et al., 2009; Wiedemann and Pfanner, 2017; Pfanner et al., 2019). In order to avoid the import of aberrant proteins into mitochondria and to ensure mitochondrial proteostasis, specialized surveillance mechanisms monitor the nuclear-encoded proteins before and during mitochondrial import (Figures 1, 2).

Interestingly, it was shown that mitochondrial precursor proteins are constantly degraded in a ubiquitin-dependent manner. For example, both the wild-type and mutant IMS protein endonuclease G (endoG) are degraded by the UPS before their import (Radke et al., 2008). However, in contrast to the mutant form, wild-type endoG can be alternatively degraded by the IMS protease Omi, supporting the idea that the proteasome plays a role in degradation of defective mitochondrial proteins prior to import. In line with this conclusion, the UPS was reported to assist the import of intermembrane proteins by constantly degrading precursor proteins before they enter mitochondria (Bragoszewski et al., 2013; Kowalski et al., 2018) (Figure 1).

In mammalian cells, quality control of membrane proteins recruited to mitochondria is provided by ubiquilins (Itakura et al., 2016; Whiteley et al., 2017), which are substrate receptors supporting proteasomal turnover. They typically possess a ubiquitin-binding UBA domain for recognition of ubiquitylated substrates and a ubiquitin-like UBL domain for proteasome targeting. Ubiquilins bind to mitochondrial proteins containing transmembrane domains, thereby preventing their aggregation (Itakura et al., 2016). As long as the substrate remains unmodified, the UBA domain of ubiquilin is bound to its own UBL domain, promoting mitochondrial import. Upon substrate ubiquitylation, the UBL domain is released, which results in targeting the mitochondrial substrate proteins for proteasomal degradation. Thus, ubiquilins exert an important triage function regulating mitochondrial import (Figure 2).

Mitochondrial targeting and protein import are governed at the mitochondrial import channel by a newly proposed MAD pathway. A subpopulation of Ubx2 binds to the TOM complex, which recruits Cdc48 to initiate degradation of partially imported proteins similarly to ERAD (Mårtensson et al., 2019) (Figure 2 and Table 1). This mechanism is termed mitochondrial protein translocation-associated degradation (mitoTAD). Interestingly, Ubx2 binding with Cdc48 depends on the co-factor Ufd1, whereas the other Cdc48 partners implicated in MAD, Vms1 and Doa1/Ufd3, are not involved in mitoTAD. However, combined deletion of Ubx2 with either Vms1 or Msp1 caused strong mitochondrial defects and an increase of ubiquitylated proteins bound to the TOM complex, suggesting redundant roles of the described mitochondrial import control pathways (Mårtensson et al., 2019). Indeed, Msp1 has been reported to function in degrading substrates from the TOM channel as well (Weidberg and Amon, 2018). Mitochondrial import stress induces the expression of Cis1, which mediates the recruitment of Msp1 to the import pore (Weidberg and Amon, 2018; Boos et al., 2019). Subsequently, Msp1 triggers proteasomal turnover of proteins clogging the TOM complex, thereby maintaining mitochondrial protein import and proteostasis (Figure 2) (Weidberg and Amon, 2018).

### Ribosome-Associated Quality Control of Mitochondrial Proteins

The first contact of mitochondrial proteins with the cytosolic proteostasis network occurs already during translation at cytosolic ribosomes. It was recently shown that stalling of ribosomes, which indicates aberrant mRNA, defective ribosome assembly, or an accumulation of nascent protein, triggers ribosome-associated quality control (RQC) (Brandman and Hegde, 2016; Joazeiro, 2019). Stalled ribosomes are sensed by the yeast RQC complex subunit Rqc2 (NEMF in mammals), recruiting the E3 ligase Ltn1 (listerin in mammals) for polyubiquitylation of the nascent peptide. The polyubiquitin chain serves as signal to attract Cdc48 and its cofactors Ufd1 and Npl4 (UFD1 and NPLOC4 in mammals), which together shuttle the polypeptide to the proteasome. In case the accessible nascent polypeptide does not contain any lysine residues, Rqc2 can induce a peptide extension by addition of a C-terminal alanine and threonine (CAT) tail. Through this elongation, more residues of the nascent chain get exposed outside of the ribosome exit tunnel, until a lysine becomes available for Ltn1 dependent ubiquitylation. In addition, CAT-tails have been reported to induce aggregation of proteins, which might act as a protective mechanism in case of RQC failure.

Since the targeting sequence of many mitochondrial proteins is at the N-terminus, import can occur either post-translationally or co-translationally (Neupert and Herrmann, 2007; Wiedemann and Pfanner, 2017). If import and translation occur simultaneously, part of the nascent polypeptide chain is sequestered inside the mitochondrial import machinery before completion of translation and release from the ribosome. Thus, in contrast to cytosolic proteins, ubiquitylation of mitochondrial proteins emerging at the ribosome might fail due to the close proximity between the ribosome and the mitochondrial translocation machinery and reduced accessibility of the co-translationally imported nascent polypeptide chain. Consequently, Rqc2 dependent CAT tailing can induce protein aggregation inside the mitochondrial matrix and subsequent degeneration of the mitochondrial respiratory capacity (Izawa et al., 2017). The formation of protein aggregates is counteracted by Vms1 (ANKZF1 in mammals) activity, which mediates the release of nascent polypeptides from the tRNA and thereby terminates CAT tail formation (Izawa et al., 2017; Rendón et al., 2018; Verma et al., 2018) (Figure 2 and Table 1).

## Mitochondrial Stress Response Mechanisms

Based on the intricate network of ubiquitin-dependent quality control mechanisms, stressed or defective mitochondria often affect UPS activity, which largely relies on the abundance and activity of the 26S proteasome (Marshall and Vierstra, 2019). Central to this regulatory relationship are reactive oxygen species (ROS), that are generated inside mitochondria via oxidative phosphorylation (Lefaki et al., 2017). Changes in ROS level have been linked to reversible disassembly of the 26S proteasome into 20S and 19S subunits. These changes in proteasomal composition and activity might reflect a protective strategy by favoring ATP/ubiquitin-independent degradation of oxidized proteins via 20S proteasomes (Wang et al., 2010; Grune et al., 2011; Livnat-Levanon et al., 2014). In *C. elegans*, mitochondrial impairment was linked to defective turnover of cytosolic UPS model substrates despite no increase in ROS, suggesting the existence of a distinct response mechanisms regulating UPS activity (Segref et al., 2014). Mitochondrial translation accuracy has been as well-linked to cytosolic proteostasis (Suhm et al., 2018). Particularly, mitochondrial ribosome mutations in yeast either improving or reducing translation accuracy showed an increased or decreased turnover of a cytosolic proteasome substrate, respectively. However, in both studies, proteasome activity was not altered in comparison to wild-type controls, suggesting that the regulation occurs upstream of proteasomal degradation (Segref et al., 2014; Suhm et al., 2018).

Interestingly, recent studies have demonstrated that specialized stress response pathways induced by mitochondrial impairment affect the UPS by regulating gene expression in the nucleus and protein translation in the cytosol (Papa and Germain, 2011; Nargund et al., 2012; Wrobel et al., 2015; Weidberg and Amon, 2018; Boos et al., 2019). Along with the well-known heat shock response (HSR) in the cytosol (Richter et al., 2010) and the unfolded protein response (UPR) in the ER (Walter and Ron, 2011), a mitochondrial UPR (UPR^mt^) has been thoroughly investigated (Münch, 2018; Shpilka and Haynes, 2018). These three stress response mechanisms are characterized by one or more signal transduction pathways that activate transcription of protective genes, encoding molecular chaperones, proteases, and UPS components. Consequently, induction of HSR, UPR, and UPR^mt^ supports proteostasis by inducing UPS function.

**FIGURE 3:**
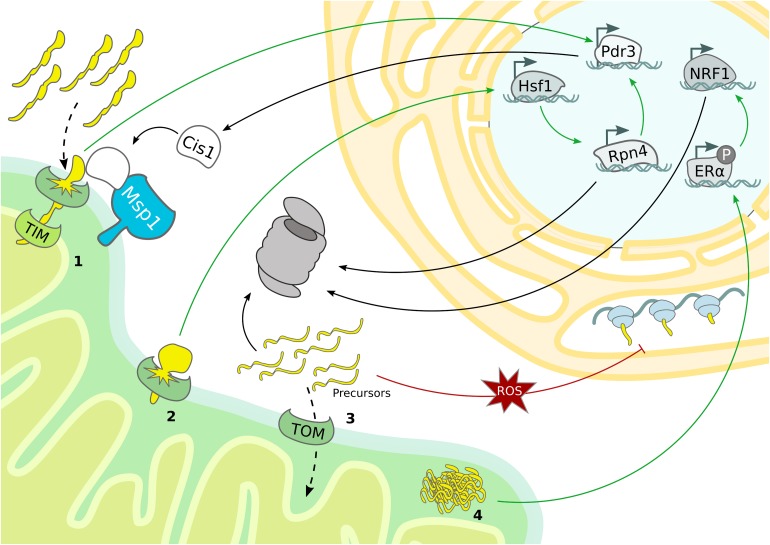
Mitochondrial stress response mechanisms. Mitochondrial stress pathways regulate nuclear gene transcription and cytosolic protein translation to sustain proteostasis. (1) Overexpression of bipartite signal-containing proteins activates the mitoCPR, which triggers Pdr3-dependent expression of MDR genes, from which Cis1 promotes the recruitment of Msp1 to the TOM complex. (2) TOM clogging activates Hsf1-dependent expression of Rpn4, which drives expression of proteasomal subunits and mitoCPR-induced Pdr3. (3) The accumulation of mitochondrial precursor proteins caused by import defects boosts proteasomal activity and depletes general protein translation in the cytosol. (4) In mammals, protein aggregation in the IMS induces proteasome activity by ligand-independent activation of ERα and subsequent upregulation of NRF1.

Even though first detected in mammalian cells (Martinus et al., 1996; Zhao et al., 2002), the UPR^mt^ mechanism has been characterized in *C. elegans* (Yoneda, 2004; Haynes et al., 2010; Nargund et al., 2012). Central to this mitochondrial stress response is the transcription factor ATFS-1, which translocates into the nucleus when mitochondrial import is blocked (Nargund et al., 2012). Although the downstream transcriptional regulation induced in case of mitochondrial stress is similar to *C. elegans*, the regulatory signaling of the mammalian UPR^mt^ emerged to be more complex and a general consensus model is still missing (Münch, 2018). Notably, a specialized mammalian UPR^mt^ was reported to enhance proteasomal activity in response to protein aggregation in the IMS (Papa and Germain, 2011). The proposed molecular mechanism is based on the ligand-independent activation of the estrogen receptor alpha (ERα), which upregulates NRF1, a transcription factor involved in the expression of proteasomal subunits (Figure 3 and Table 1) (Papa and Germain, 2011). Accordingly, the *C. elegans* NRF homolog SKN-1 has also been identified as a downstream target of the UPR^mt^ (Nargund et al., 2012). In yeast, increased proteasome activity was detected in consequence of mitochondrial import defects, indicating an UPR activated by the mistargeting of proteins (UPR^am^) (Wrobel et al., 2015). Thus, defective mitochondrial import generates an accumulation of misfolded proteins in the cytosol, which aggravates proteasomal assembly and proteolytic activity (Figure 3).

The use of deep transcriptome sequencing, combined with proteomics allowed to monitor transcriptional and translational changes over time after ‘clogging’ of the TOM complex in yeast. Under these conditions the heat shock responsive transcription factor Hsf1 is activated and triggers the expression of molecular chaperones. Interestingly, one Hsf1-dependent target gene encodes the transcription factor Rpn4, which specifically upregulates proteasomal subunits, constituting a second layer of response that is activated upon prolonged stress (Boos et al., 2019). In addition to Hsf1 activation, the expression of nuclear encoded respiratory chain subunits is downregulated, which might serve to reduce the mitochondrial import load (Figure 3). Another transcriptional stress response activated by import defects is the mitochondrial compromised protein import response (mitoCPR) (Weidberg and Amon, 2018). Here, import inhibition caused by overexpression of bipartite signal-containing proteins, which are normally inserted into the IMS, provokes expression of multi drug resistance (MDR) response genes by the transcription factor Pdr3. One of the most upregulated genes is Cis1, which is recruited to the OMM and, by interacting with Tom70 and Msp1, supports the Msp1 and proteasome-dependent degradation of non-imported mitochondrial precursor proteins. Interestingly, the correct functionality of this pathway is fundamental for cell survival upon defective mitochondrial import but not under normal growth conditions (Figure 3 and Table 1) (Weidberg and Amon, 2018).

Although a general transcriptional program has not been identified yet, an intricate cooperation of the different quality control pathways becomes evident. For example, Rpn4 is not only initiated upon TOM “clogging,” but also in import-defective yeast mutants related to UPR^am^ (Wrobel et al., 2015; Boos et al., 2019). However, in contrast to clogging the TOM channel (Boos et al., 2019), import-defects do not induce the expression of proteasome subunits but of proteasome assembly chaperones (Wrobel et al., 2015). These discrepancies suggest that Rpn4 might be activated in case of mitochondrial defects, but probably distinct transcriptional programs characterize specific types of import-related stress. Moreover, the Pdr3-dependent transcriptional induction of Cis1 upon accumulation of bipartite signal-containing proteins also requires Rpn4 (Figure 3) (Boos et al., 2019).

An additional cellular strategy to avoid UPS overload under prolonged mitochondrial stress conditions is based on suppression of protein translation both inside and outside of the organelle (Münch, 2018; Samluk et al., 2018; Shpilka and Haynes, 2018). The predominant mechanism of cytosolic translation inhibition is mediated by phosphorylation of the eukaryotic transition initiation factor 2 alpha (eiF2α), which prevents formation of the translation initiation complex (Sonenberg and Hinnebusch, 2009). However, oxidative stress generated upon dysfunctional mitochondrial import was recently reported to reduce cytosolic translation independently of eiF2α phosphorylation. For instance, the translation machinery can be directly modulated by the redox status of proteins participating in translation (Figure 3) (Topf et al., 2018). Besides reducing global protein translation, mitochondrial import defects have also been proposed to induce translation of particular mRNAs (Wang and Chen, 2015; Topf et al., 2016). Specialized translation of stress-related transcription factors has already been described as a downstream event of eiF2α phosphorylation, which therefore trigger numerous stress responses, including the UPR^mt^ (Fiorese et al., 2016; Pakos-Zebrucka et al., 2016; Quirós et al., 2017).

Even though a common mechanism has not been identified yet, it is obvious that diverse, overlapping mitochondrial signaling pathways are activated to support proteostasis and cellular survival. These pathways influence gene expression to adapt mitochondrial and cytosolic protein degradation pathways under mitochondrial stress conditions.

## Conclusion

**FIGURE 4:**
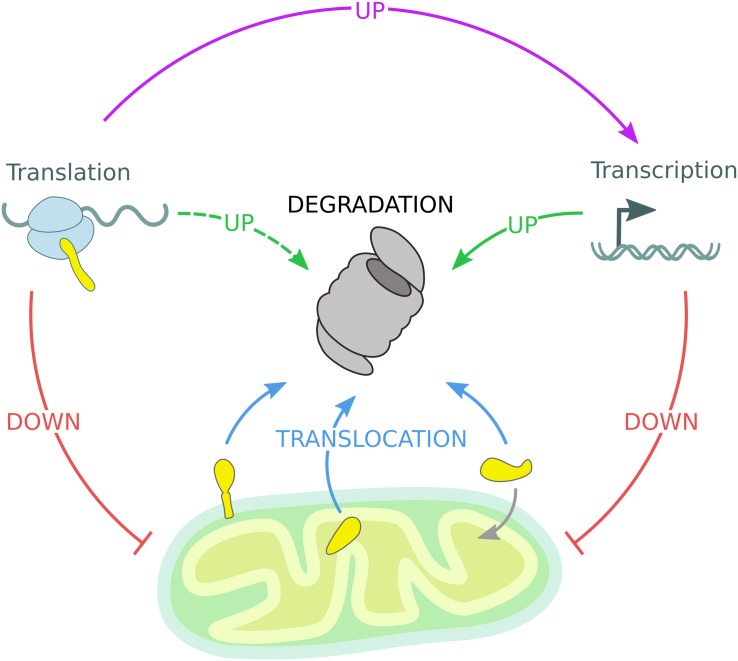
Mitochondrial quality control governed by the UPS. The mitochondrial proteome is regulated by constant degradation of mitochondrial proteins, which are sequestered from the organelle and translocated (blue arrows) to the 26S proteasome for degradation. In addition, mitochondrial precursor proteins are degraded by the 26S proteasome if not efficiently imported into mitochondria. Upon mitochondrial stress, transcription and translation of mitochondrial genes are diminished, reducing the substrate load of both the mitochondrial import machinery and the 26S proteasome (red arrows). Moreover, transcription of protective genes is induced to enhance UPS activity (solid green arrow). Stress-induced inhibition of global protein translation triggers the expression of specialized transcription factors responsible for the activation of inducible stress response programs mentioned before (violet arrow), while other gene products might support UPS function (dashed green arrow).

Each cellular sub-compartment is equipped with specialized quality control machineries, which are intricately connected and cross-communicate. The reported studies support the idea that ubiquitin-dependent mitochondrial quality control pathways efficiently adapt in response to environmental and metabolic changes. However, in case of acute stress conditions, specialized response programs are induced to adjust the UPS capacity and thereby restore organellar proteostasis (Pakos-Zebrucka et al., 2016; Braun and Westermann, 2017; D’Amico et al., 2017; Pickles et al., 2018; Andréasson et al., 2019; Zheng et al., 2019; Escobar-Henriques et al., 2020). Besides understanding the mechanistic details of individual pathways, the regulation of cell-type specific and organismal composition of mitochondrial quality control need to be further addressed. Mechanistically, ubiquitin-dependent mitochondrial proteostasis follows a series of common regulatory events: stress sensing, substrate targeting and modification, protein translocation, and proteasomal degradation. Mitochondrial quality control is further regulated by nuclear gene transcription and cytosolic protein translation events (Figure 4). Overall, these proteostasis strategies are highly adaptive and can efficiently and dynamically modulate the stability of the mitochondrial proteome according to cellular needs.

## Future Perspectives

Regulation of mitochondrial quality control by the UPS emerged to be conserved in all eukaryotes, involving Cdc48/p97 and the 26S proteasome. However, mechanistic details on substrate selection and ubiquitin ligases remain largely unclear. In contrast to proteasomal degradation of OMM proteins, little is known about the turnover of intra-mitochondrial proteins. Especially how mitochondrial substrates are retro-translocated from the different inner mitochondrial compartments into the cytosol is of central importance for understanding the regulation of MAD. Conversely, a novel functional role of the mitochondrial translocation machinery has been identified, which seems to import cytosolic aggregation-prone proteins into mitochondria for efficient degradation (Ruan et al., 2017). A more detailed view on the protein shuttling between mitochondria and cytosol will extend our current view on the cellular mechanisms dedicated to proteostasis maintenance and the reciprocal role of mitochondrial and cytosolic proteolytic systems.

Besides the high degree of mechanistic similarities between the MAD and ERAD pathways, mitochondrial quality control is further defined by functional mitochondria-ER interactions. For example, substrates extracted from the OMM by Msp1 are targeted to the ER for subsequent proteasomal degradation (Dederer et al., 2019; Matsumoto et al., 2019). Moreover, mitochondrial precursor proteins have been identified to associate with the ER membrane before being imported. The recently proposed ER-surface mediated targeting (ER-SURF) model describes the association of mitochondrial precursor proteins with the ER membrane and their rerouting to mitochondria by the ER-localized chaperone Djp1 (Hansen et al., 2018). Thus, the interaction between mitochondria and ER might play a conceptual role in protein quality control of mitochondrial proteins.

Mitochondrial impairments have been associated to several pathologies not only limited to metabolic diseases or myopathies, but including cancer (Vyas et al., 2016; Denisenko et al., 2019), pulmonary hypertension (Chen et al., 2019) and neurodegenerative disorders such as Alzheimer’s and Parkinson’s disease (Kim and Mook-Jung, 2019; Tapias, 2019). Thus, further understanding of mitochondrial surveillance mechanisms might help to establish therapeutic interventions for treatment of mitochondrial pathologies. The multiple layers of mitochondrial regulation that can lead to disease progression if defective makes mitochondrial quality control a challenging but exciting research field, which is more and more integrated in the context of specialized cellular pathways, from basic research to clinical studies.

## Author Contributions

All authors conceived, wrote the manuscript, and critically revised the manuscript. SR designed the figures.

## Conflict of Interest

The authors declare that the research was conducted in the absence of any commercial or financial relationships that could be construed as a potential conflict of interest.
